# IRAG2 Interacts with IP_3_-Receptor Types 1, 2, and 3 and Regulates Intracellular Ca^2+^ in Murine Pancreatic Acinar Cells

**DOI:** 10.3390/ijms222413409

**Published:** 2021-12-14

**Authors:** Sally Prüschenk, Michael Majer, Rainer Schreiber, Jens Schlossmann

**Affiliations:** 1 Department of Pharmacology and Toxicology, Institute of Pharmacy, University of Regensburg, 93040 Regensburg, Germany; Sally.Prueschenk@chemie.uni-regensburg.de (S.P.); Michael.Majer@chemie.uni-regensburg.de (M.M.); 2 Institute of Physiology, University of Regensburg, 93040 Regensburg, Germany; Rainer.Schreiber@vkl.uni-regensburg.de

**Keywords:** Ca^2+^ signaling, IP_3_R, IRAG, IRAG1, IRAG2, Jaw1, LRMP, pancreas, pancreatic acinar cells

## Abstract

The inositol 1,4,5-triphosphate receptor-associated 2 (IRAG2) is also known as Jaw1 or lymphoid-restricted membrane protein (LRMP) and shares homology with the inositol 1,4,5-triphosphate receptor-associated cGMP kinase substrate 1 (IRAG1). IRAG1 interacts with inositol trisphosphate receptors (IP_3_ receptors /IP_3_R) via its coiled-coil domain and modulates Ca^2+^ release from intracellular stores. Due to the homology of IRAG1 and IRAG2, especially in its coiled-coil domain, it is possible that IRAG2 has similar interaction partners like IRAG1 and that IRAG2 also modulates intracellular Ca^2+^ signaling. In our study, we localized IRAG2 in pancreatic acinar cells of the exocrine pancreas, and we investigated the interaction of IRAG2 with IP_3_ receptors and its impact on intracellular Ca^2+^ signaling and exocrine pancreatic function, like amylase secretion. We detected the interaction of IRAG2 with different subtypes of IP_3_R and altered Ca^2+^ release in pancreatic acinar cells from mice lacking IRAG2. IRAG2 deficiency decreased basal levels of intracellular Ca^2+^, suggesting that IRAG2 leads to activation of IP_3_R under unstimulated basal conditions. Moreover, we observed that loss of IRAG2 impacts the secretion of amylase. Our data, therefore, suggest that IRAG2 modulates intracellular Ca^2+^ signaling, which regulates exocrine pancreatic function.

## 1. Introduction

Pancreatic acinar cells have been a model for the investigation of Ca^2+^ signaling and enzymatic secretion over a long period. Changes in Ca^2+^ signaling in these cells can lead to the altered secretion of digestive enzymes and are associated with severe pancreatic diseases like acute pancreatitis due to excessive Ca^2+^ release from intracellular stores [1].

Inositol trisphosphate receptors (IP_3_ receptors /IP_3_R) are intracellular Ca^2+^ release channels that play an important role in Ca^2+^ signaling. The effect of different subtypes of IP_3_ receptors and the extent of their contribution to Ca^2+^ signaling and Ca^2+^ fluctuation is still widely unknown [2]. In pancreatic acinar cells, it is described that IP_3_R2 and IP_3_R3 are the major Ca^2+^ release channels that are responsible for exocrine secretion [3]. However, all of the three subtypes of IP_3_R are expressed at the secretory pole of pancreatic acinar cells [4]. Upon muscarinic stimulation with pancreatic secretagogues, phospholipase C is activated, which cleaves phosphatidylinositol-4,5-bisphosphate (PIP_2_) into the second messenger inositol 1,4,5-trisphosphate (IP_3_). IP_3_ binds to IP_3_ receptors and induces Ca^2+^ release from intracellular stores [5].

The inositol 1,4,5-triphosphate receptor-associated 2 (IRAG2), also known as Jaw1 or lymphoid-restricted membrane protein (LRMP), is a type ІІ membrane protein that is localized to the cytoplasmic face of the endoplasmic reticulum (ER) and contains a coiled-coil domain and a COOH-terminal hydrophobic anchor domain [6,7]. Especially in the coiled-coil domain, IRAG2 reveals a homology of 44% with the inositol 1,4,5-triphosphate receptor-associated cGMP kinase substrate 1 (IRAG1) [8,9,10]. IRAG1 interacts with IP_3_R via its coiled-coil domain and regulates Ca^2+^ release from intracellular stores [11,12]. It is shown that IRAG2 interacts with IP_3_R3 via its coiled-coil domain in the COS7 heterologous expression system and with IP_3_R2 in intestinal tuft cells [9,13].

Expression of IRAG2 was found in B-cell lines, T-cell lines, spleen, thymus, and intestinal tuft cells [6,9,13,14]. IRAG2 was also detected in sinoatrial nodes and in sweet, bitter, and umami taste-responsive cells [8,9].

It has been previously described that IRAG2 expression is regulated by p53 in intestinal tuft cells and that IRAG2 ensures Ca^2+^ flux in these cells, which is a critical trigger for IL-25 release in response to parasitic infections [13]. A previous study also reported that IRAG1 and IRAG2 are isoform-specific interaction partners and modulators of HCN4, with opposing effects on these channels [8]. In sweet, bitter, and umami taste-responsive cells, IRAG2 is co-expressed with IP_3_R3 [9].

Based on these data, it is possible that IRAG2 also interacts with IP_3_R in other tissues and modulates intracellular Ca^2+^ signaling. However, physiological and molecular functions of IRAG2, especially its possible role as a modulator of the IP_3_-Ca^2+^ cascade, are still widely unknown. In the pancreas, no information exists about the expression pattern or function of IRAG2 as a modulator of intracellular Ca^2+^ signaling. In our study, we analyzed the expression pattern and interaction of IRAG2 with different IP_3_ receptors in the murine pancreas as well as its role in Ca^2+^ signaling and exocrine pancreatic function.

## 2. Results

### 2.1. Expression of IRAG2 in Pancreatic Acinar Cells

To examine the expression and localization of IRAG2, we generated a lacZ×IRAG2-KO mouse line (genotype: *lacZ* *× Irag2*^−/−^), expressing β-galactosidase as a reporter for IRAG2 as previously described [13]. In addition, to study the function of IRAG2, we generated an IRAG2-KO mouse line (genotype: *Irag2*^−/−^) without β-galactosidase (Figure S1). Expression of IRAG2 in whole murine pancreas tissue and pancreatic acinar cells was analyzed via Western blotting and X-Gal staining using lacZ×IRAG2-KO mice, IRAG2-KO mice and their WT littermates (IRAG2-WT; genotype: *Irag2^+/+^, Irag2^fl/fl^*).

**Figure 1:**
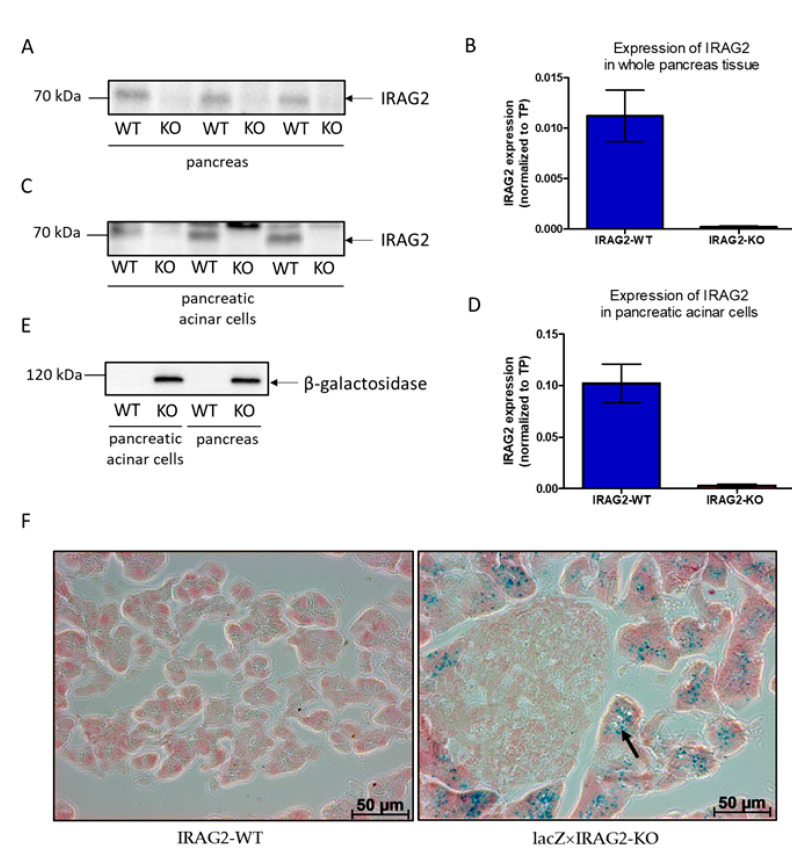
Expression and localization of IRAG2 in the murine pancreas. (**A,C**) Western blot analysis showed expression of IRAG2 protein in whole pancreas tissue (**A**) and isolated pancreatic acinar cells (**C**) of IRAG2-WT mice (WT), but no protein was detected in IRAG2-KO mice (KO). (**B**) IRAG2 expression in whole pancreas tissue of IRAG2-WT and IRAG2-KO mice normalized to total protein (TP) (WT: *n* = 12; KO: *n* = 12). (**D**) IRAG2 expression in pancreatic acinar cells of IRAG2-WT and IRAG2-KO mice normalized to total protein (TP) (WT: *n* = 6; KO: *n* = 6). (**E**) Western blot analysis indicated expression of β-galactosidase as a reporter for IRAG2 in whole pancreas and isolated pancreatic acinar cells of lacZ×IRAG2-KO mice (KO) but not in IRAG2-WT mice (WT). (**F**) Localization of β-galactosidase by X-Gal staining on pancreas cryosections from lacZ×IRAG2-KO and IRAG2-WT mice. Expression was detected in pancreatic acinar cells (indicated by arrow) but not in islets of Langerhans from lacZ×IRAG2-KO pancreata. No expression of β-galactosidase was detected in IRAG2-WT pancreata. In the graphs, mean ± SEM are shown. Images of total protein (TP) are shown in Figure S4.

Western blot analysis showed expression of IRAG2 in whole pancreas tissue of IRAG2-WT mice but not in IRAG2-KO mice (Figure 1A,B). To investigate the cell types, which express IRAG2 in the murine pancreas, X-Gal staining for β-galactosidase activity was performed on pancreata from lacZ×IRAG2-KO mice and IRAG2-WT mice, where β-galactosidase acts as a reporter for IRAG2. Staining of β-galactosidase was detected in pancreatic acinar cells of lacZ×IRAG2-KO mice but not in IRAG2-WT mice (Figure 1F). No β-galactosidase activity was detected in islets of Langerhans (Figure 1F) or pancreatic ducts (Figure S2). To confirm these data, Western blot analysis of isolated pancreatic acinar cells was performed, and IRAG2 was detected in acinar cells of IRAG2-WT but not in IRAG2-KO (Figure 1C,D). Furthermore, expression of β-galactosidase was analyzed in lysates using an anti-β-galactosidase-antibody and was detected in whole pancreas tissue and pancreatic acinar cells from lacZ×IRAG2-KO mice, but not in IRAG2-WT mice (Figure 1E). Taken together, these data revealed IRAG2 expression in murine pancreas and localization in pancreatic acinar cells.

As IRAG2 has been previously described as a homolog of IRAG1 [8,9,10], we investigated if IRAG1 is also expressed in whole pancreas tissue or pancreatic acinar cells. Detection of IRAG1 was neither seen in pancreatic acinar cells nor in whole pancreas tissue of IRAG2-WT or IRAG2-KO mice (Figure S3). As a positive control for IRAG1 expression, platelets of IRAG1-WT and as a negative control platelets of IRAG1-KO mice were applied since expression of IRAG1 in platelets has been described before [15].

### 2.2. Interaction of IRAG2 with IP_3_R Types 1, 2, and 3 and Regulation of IP_3_R Expression

**Figure 2:**
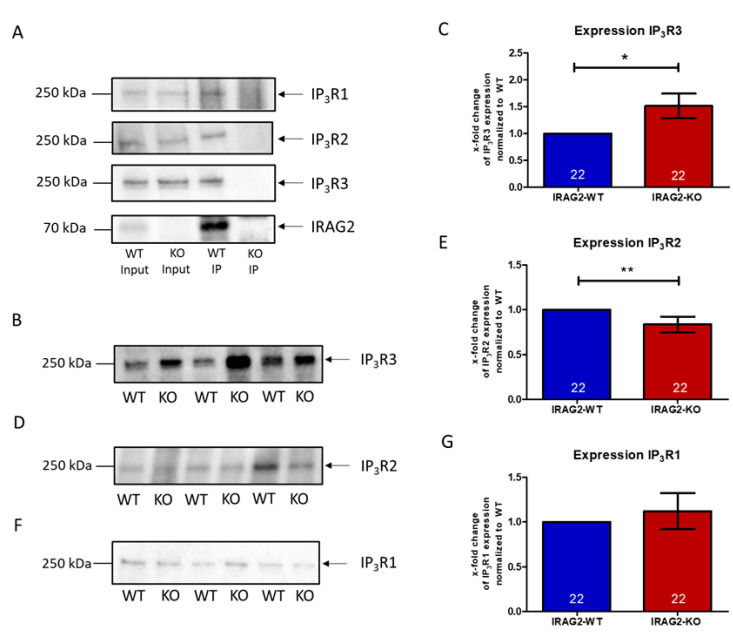
Interaction of IRAG2 with IP_3_ receptor types 1, 2, and 3 (**A**) and expression of different IP_3_ receptors in pancreata from IRAG2-WT (WT) and IRAG2-KO mice (KO) (**B**–**G**). (**A**) After coimmunoprecipitation of pancreas lysates from IRAG2-KO (KO) and IRAG2-WT (WT) mice with IRAG2 antibody (1 µg), proteins were detected by Western blot. For coimmunoprecipitation, 1500 µg and for input 70 µg protein was used. (**B**,**D**,**F**) Protein expression of IP_3_R3, IP_3_R2, and IP_3_R1 was analyzed by Western blot and normalized to total protein (TP) for quantification. (**B**,**C**) Representative Western blot of IP_3_R3 showed a significant increase of IP_3_R3 expression in pancreata of IRAG2-KO (KO) mice compared to IRAG2-WT (WT) mice. (**D**,**E**) Analysis of IP_3_R2 showed a significant decrease of IP_3_R2 expression in IRAG2-KO (KO) compared to IRAG2-WT (WT). (**F**,**G**) IP_3_R1 expression was not altered between IRAG2-WT (WT) and IRAG2-KO (KO). (**C**,**E**,**G**) Statistical analysis of the different IP_3_ receptors (**B**,**D**,**F**). Results are given as x-fold change of expression normalized to IRAG2-WT. Images of total protein (TP) are shown in Figure S5. Numbers of analyzed lysates are indicated in the graphs, and the mean ± SEM is shown by bars. Significant differences are indicated by (*) (*p* < 0.05) and (**) (*p* < 0.01).

Because of its homology to IRAG1, especially in its coiled-coil domain [8,9,10], it might be possible that IRAG2 has similar interaction partners like IRAG1. To test whether IRAG2 interacts with different IP_3_-receptor subtypes in murine pancreas, IRAG2 was immunoprecipitated by IRAG2 antibody, and interaction partners were analyzed via Western blot. All three subtypes of IP_3_ receptors, IP_3_R types 1, 2, and 3, were detected as coimmunoprecipitated interaction partners (Figure 2A). To see whether a knockdown of IRAG2 leads to different expressions of IP_3_R subtypes, we analyzed the expression of IP_3_R1, IP_3_R2, and IP_3_R3 in pancreata from IRAG2-WT and IRAG2-KO mice. Expression was normalized to total protein (Figure S3) and calculated as x-fold change of IRAG2-KO expression normalized to IRAG2-WT. Expression of IP_3_R3 was significantly higher in IRAG2-KO mice compared to IRAG2-WT (Figure 2B,C), and expression of IP_3_R2 (Figure 2D,E) was significantly lower in pancreata from IRAG2-KO. IP_3_R1 expression (Figure 2F,G) was not altered between IRAG2-KO and IRAG2-WT pancreas.

### 2.3. Intracellular Ca^2+^ Regulation in IRAG2-KO Pancreatic Acinar Cells

**Figure 3:**
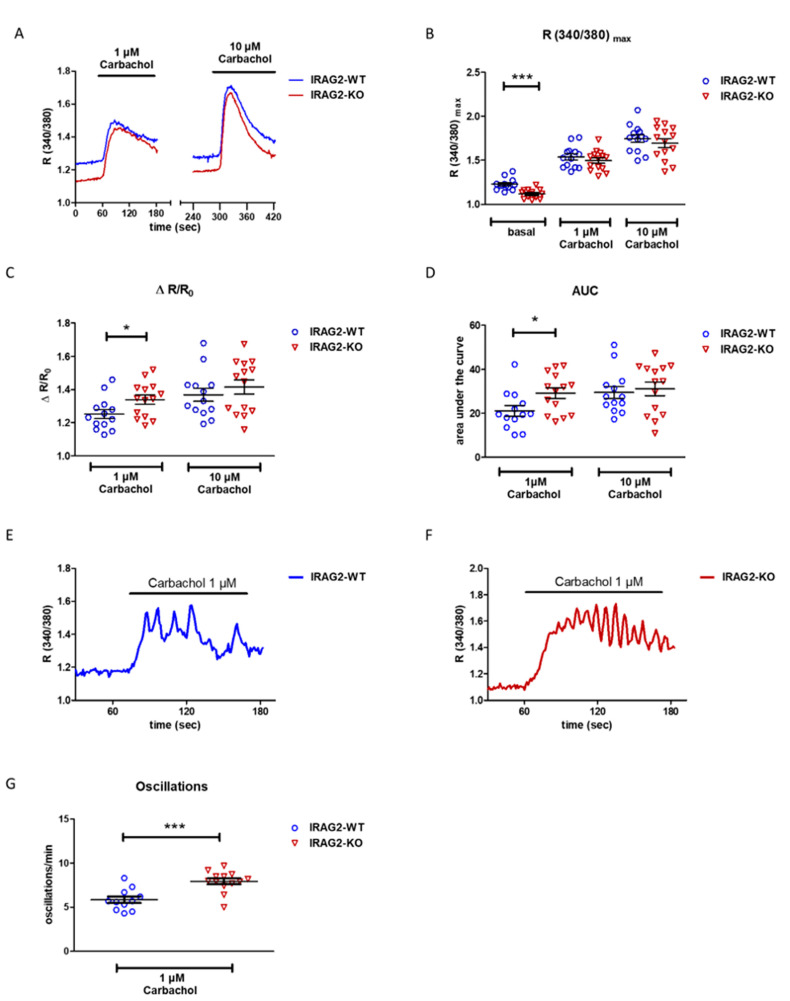
Regulation of intracellular calcium in isolated pancreatic acinar cells from IRAG2-WT and IRAG2-KO mice. (**A**) Stimulation of calcium release in Fura-2-AM loaded cells (2 µM Fura-2-AM; 90 min in the dark at room temperature (RT)) with 1 µM carbachol and 10 µM carbachol. Cells were stimulated with different carbachol concentrations for 2 min; between stimulations, cells were allowed to recover for 2 min under perfusion with Ringer´s solution. (**B**) Statistical evaluation revealed lower basal Ca^2+^ levels in IRAG2-KO pancreatic acinar cells compared to IRAG2-WT cells (WT: *n* = 13 experiments from 5 animals; KO: *n* = 14 experiments from 5 animals). (**C**) IRAG2-KO cells showed a higher release of calcium x-fold of basal level (R/R_0_) compared to IRAG2-WT when stimulated with 1 µM carbachol, but not with 10 µM carbachol (WT: *n* = 13 experiments from 5 animals, KO: *n* = 14 experiments from 5 animals). (**D**) Area under the curve (AUC) was calculated from release curves and was increased in IRAG2-KO compared to IRAG2-WT upon stimulation with 1 µM carbachol, but not with 10 µM carbachol (WT: *n* = 13 experiments from 5 animals, KO: *n* = 14 experiments from 5 animals). (**E**,**F**) Oscillations in IRAG2-WT (**E**) and IRAG2-KO (**F**) were detected when stimulating the cells with 1 µM carbachol. (**G**) Oscillations/min were counted, statistical evaluation showed a higher number of oscillations per min in IRAG2-KO pancreatic acinar cells compared to IRAG2-WT (WT: *n* = 11 oscillating cells from 5 animals; KO: *n* = 13 oscillating cells from 5 animals). Circles indicate the individual value of each experiment in IRAG2-WT, and triangles show individual values for each experiment from IRAG2-KO. Graphs (**B**,**C**,**D**,**G**) are shown as mean ± SEM, and significant differences are indicated as (*) (*p* < 0.05), and (***) (*p* < 0.001).

IRAG1 interacts with IP_3_ receptors and modulates intracellular Ca^2+^ release in smooth muscle cells [11,12]. Since IRAG2, as a homolog of IRAG1 [8,9,10], interacted with different subtypes of IP_3_ receptors in the murine pancreas (2.2.) and due to localization of IRAG2 in pancreatic acinar cells (2.1.), we isolated the acinar cells of the exocrine pancreas to see whether knockdown of IRAG2 impacts Ca^2+^ signaling. Isolated, Fura2-AM loaded acinar cells were equilibrated with Ringer’s solution for 2 min. Following that, cells were first stimulated with 1 µM carbachol, and after 2 min recovery time under perfusion with Ringer´s solution, cells were stimulated with 10 µM carbachol to evoke Ca^2+^ release from the endoplasmic reticulum (ER). The Ca^2+^ transients were monitored by the ratio of Fura-2 fluorescence (R_340/380_). Basal, unstimulated ratio (R_340/380_) was highly significantly lower in IRAG2-KO cells compared to IRAG2-WT cells, indicating that basal intracellular Ca^2+^ was decreased in IRAG2-KO (Figure 3A,B). Upon stimulation with 1 µM carbachol and 10 µM carbachol, there was a tendency for a slightly decreased Ca^2+^ release in IRAG2-KO, indicated by a decreased R_340/380_, which was not significant (Figure 3B). To normalize the quantity of released Ca^2+^, the maximum ratio upon stimulation with 1 µM or 10 µM carbachol was calculated as x-fold of basal ratio (∆ R/R_0_). IRAG2-KO showed a higher ∆ R/R_0_ than IRAG2-WT after stimulation with 1 µM, whereas 10 µM carbachol had no effect. This implied that relatively more Ca^2+^ was released upon stimulation with 1 µM carbachol in IRAG2-KO compared to IRAG2-WT (Figure 3C). The area under the curve (AUC) was calculated over a stimulation period of 2 min when cells were stimulated with 1 µM or 10 µM carbachol. This value allows the comparison of the amount of released Ca^2+^ between IRAG2-WT and IRAG2-KO cells. Calculated AUC was significantly higher in IRAG2-KO than in IRAG2-WT when stimulating with 1µM, but not with 10 µM carbachol, indicating that higher amounts of Ca^2+^ were released upon stimulation in IRAG2-KO (Figure 3D). The slope of the release curve was calculated to assess velocity of Ca^2+^ release. Slope was not altered between IRAG2-WT and IRAG2-KO neither on stimulation with 1 µM nor on 10 µM carbachol (Figure S6A). To evaluate stimulatability of cells with 10 µM carbachol after treating them with 1 µM carbachol before, maximal ratio R_340/380_ on stimulation with 10 µM carbachol was calculated x-fold of maximal ratio R_340/380_ on stimulation with 1 µM carbachol. Between IRAG2-WT and IRAG2-KO, no differences in stimulatability of cells upon treatment with higher carbachol concentrations were seen when stimulating with lower concentrations before (Figure S6B).

At lower concentrations of carbachol, pancreatic acinar cells show Ca^2+^ oscillations. This phenomenon is achieved through the release of Ca^2+^ from intracellular stores and ATP-dependent Ca^2+^ reuptake, leading to oscillating signals [16,17]. We observed Ca^2+^ oscillations in IRAG2-WT (Figure 3E) and IRAG2-KO (Figure 3F) when treating the cells with 1 µM carbachol. Oscillations per min were counted and are significantly higher in IRAG2-KO cells than in IRAG2-WT cells (Figure 3G), showing that the frequency of oscillations was higher in IRAG2-KO than in IRAG2-WT cells.

### 2.4. Amylase Content and Release in IRAG2-WT and IRAG2-KO Pancreatic Acinar Cells

**Figure 4:**
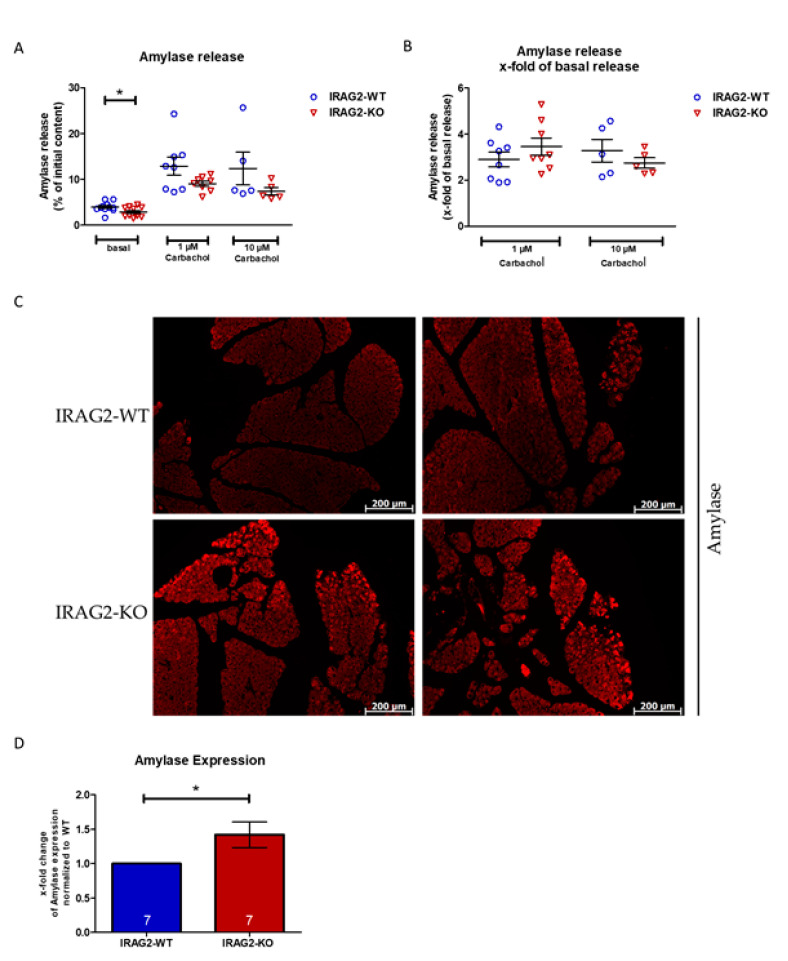
Amylase release in pancreatic acinar cells and Amylase content in whole murine pancreas. (**A**,**B**) Release of amylase was stimulated in isolated pancreatic acinar cells for 30 min using 1 µM or 10 µM carbachol or incubation buffer as control for basal release. (**A**) IRAG2-KO cells revealed a significant lower basal amylase secretion compared to IRAG2-WT (for 1 µM carbachol: WT: *n* = 8, KO: *n* = 8; for 10 µM carbachol: WT: *n* = 5, KO: *n* = 5). (**B**) Amylase release was calculated as x-fold of basal release where IRAG2-KO cells manifested a tendency for a higher amylase release x-fold of basal release upon stimulation with 1 µM carbachol but not for stimulation with 10 µM carbachol (for 1 µM carbachol: WT: *n* = 8, KO: *n* = 8; for 10 µM carbachol: WT: *n* = 5, KO: *n* = 5). (**C**) Representative immunohistochemistry of amylase in paraffine embedded pancreata of IRAG2-KO and IRAG2-WT mice showed a higher expression of amylase in IRAG2-KO pancreata compared to IRAG2-WT (50× magnification). (**D**) Quantification of amylase expression by detecting the fluorescence and statistical evaluation. Expression of amylase in IRAG2-KO pancreata was normalized to IRAG2-WT, and results were given as x-fold change of amylase expression in IRAG2-KO normalized to IRAG2-WT. Circles in the graphs indicate the individual value of each experiment in IRAG2-WT, and triangles show individual values for each experiment from IRAG2-KO. Mean ± SEM are indicated by bars in the graphs, and significant differences are shown as (*) (*p* < 0.05).

The release of Ca^2+^ from intracellular stores in response to secretagogues triggers the exocytosis of zymogen granules and consequently the release of amylase [1,18,19]. We stimulated dispersed acini with 1 µM or 10 µM carbachol for 30 min and measured secreted pancreatic amylase to evaluate the exocrine pancreatic function since IRAG2 appears to regulate Ca^2+^ signaling in pancreatic acinar cells (2.3.). IRAG2-KO pancreatic acinar cells showed a reduced basal, unstimulated amylase release compared to IRAG2-WT cells over a stimulation period of 30 min (Figure 4A). When stimulating the cells with 1 µM or 10 µM carbachol, the maximum amount of secreted amylase during 30 min was lower in IRAG2-KO than in IRAG2-WT, but not significantly (Figure 4A). Release of amylase calculated as x-fold of basal amylase release revealed a not significant tendency for an enhanced amylase secretion in IRAG2-KO compared to IRAG2-WT upon stimulation with 1 µM carbachol, but not for 10 µM carbachol (Figure 4B). The activity of amylase was examined in pancreas lysates and serum probes from IRAG2-WT and IRAG2-KO and showed no altered amylase activity between IRAG2-KO and IRAG2-WT neither in pancreas lysates (Figure S7A) nor in serum probes (Figure S7B).

To compare the total amount of amylase in exocrine pancreas tissue, immunohistochemical analysis of amylase in whole pancreas tissue was performed. IRAG2-KO showed an increased amount of amylase in pancreas tissue compared to IRAG2-WT (Figure 4C). The amount of amylase was quantified by detecting the fluorescence in IRAG2-KO and IRAG2-WT pancreata. Fluorescence and therefore quantity of expressed amylase in IRAG2-KO was normalized to IRAG2-WT, and results were given as x-fold change of amylase expression in IRAG2-KO normalized to IRAG2-WT (Figure 4D).

Since the amount of amylase was increased in IRAG2-KO pancreas tissue, hematoxylin-eosin (HE) staining was performed to compare the morphology of pancreata and the amount of zymogen granules in IRAG2-KO and IRAG2-WT mice. HE staining revealed no differences in morphology or zymogen granules between IRAG2-WT and IRAG2-KO exocrine pancreatic tissue (Figure S8). Images of dispersed pancreatic acinar cells also did not show any differences between isolated cells from IRAG2-WT and IRAG2-KO (Figure S9).

Deficiency in exocrine pancreatic function can lead to underdigestion of food and reduced body weights [3]. To analyze if IRAG2-KO mice show any abnormalities in body weight, mice were weighed starting day 9 after birth until day 47 after birth. Before weaning, no differences in body weights between IRAG2-WT and IRAG-KO mice were observed (Figure S10A,B,C). Some days after weaning (day 33–35), IRAG2-KO mice showed a slightly reduced body weight, which normalized until day 45 after birth (Figure S10A,D,E). After postnatal day 45, no differences in body weights between IRAG2-WT and IRAG2-KO were seen (Figure S10A,E).

## 3. Discussion

IRAG2 is a type II membrane protein that has been firstly described by Behrens et al. [6]. It is expressed particularly in lymphoid tissues [6,14], but expression can also be found in intestinal tuft cells [13], in sinoatrial nodes [8], and in sweet, bitter, and umami taste-responsive cells [9]. According to the Human Protein Atlas (www.proteinatlas.org), protein expression, as well as mRNA expression of IRAG2, is detectable in the human exocrine pancreas [20]. In our study, we demonstrate that IRAG2 is expressed in the murine pancreas and localized in acinar cells of the exocrine pancreas. This localization suggests that IRAG2 might be involved in exocrine pancreatic function, e.g., in exocrine secretion. These findings correlate with the data from the Human Protein Atlas for expression of IRAG2 in the human pancreas, mainly in the exocrine glandular cells. The data from the Human Protein Atlas also indicate a weak expression of IRAG2 in human pancreatic duct cells [20]. However, we could not detect the expression of IRAG2 in murine pancreatic duct cells. A possible function of IRAG2 in the human pancreatic duct cells and in human pancreatic acinar cells is unknown.

IRAG2 reveals a homology of 44% with IRAG1 in its coiled-coil domain [8,9,10]. The coiled-coil domain of IRAG1 is essential for its interaction with the IP_3_R1 [11]. The homology of IRAG1 and IRAG2 raises the possibility that IRAG2 also interacts with IP_3_R in different murine tissues. It is already described that IRAG2 interacts with IP_3_R3 via its coiled-coil domain in the COS7 heterologous expression system and is co-expressed with IP_3_R3 in sweet, bitter, and umami taste-responsive cells [9]. In intestinal tuft cells, the interaction of IP_3_R2 and IRAG2 was reported [13]. In the pancreas, all subtypes of IP_3_ receptors are expressed, and in acinar cells, all IP_3_ receptor subtypes are localized to the secretory pole of the acinus [4]. Our data demonstrate that IRAG2 interacts with IP_3_ receptor types 1, 2, and 3 in the murine pancreas. This leads to the assumption that IRAG2 is also expressed at the secretory pole of the acinus. In the Human Protein Atlas, immunohistochemical analysis of IRAG2 expression in human pancreata shows the distribution of IRAG2, particularly at the apical pole of the acinus [20], which would confirm the thesis that IRAG2 is also expressed at the secretory pole of the acinar cells in the murine pancreas. Moreover, to our knowledge, this is the first time that an interaction of IRAG2 with the IP_3_R subtype 1 is also shown.

IP_3_R type 1 is highly expressed in brain tissue and plays a role in higher brain functions such as behavior, learning, and memory [21,22,23,24]. Its role in pancreatic acinar cells is widely unknown. Expression of IP_3_R type 1 is reported to be rather low compared to IP_3_R2 and IP_3_R3 in the pancreas [25]. IP_3_ receptor types 2 and 3 have been shown to play an important role in exocrine secretion. Double knockouts of IP_3_R2 and IP_3_R3 reveal exocrine dysfunction, and Ca^2+^ signaling is severely decreased in pancreatic acinar cells. Therefore, IP_3_R2 and IP_3_R3 are the major Ca^2+^ release channels in pancreatic acinar cells that mediate secretagogue-induced exocrine pancreatic secretion [3]. In our study, we observed an upregulation of IP_3_R3 and a downregulation of IP_3_R2 expression in pancreata from IRAG2-KO compared to IRAG2-WT, whereas expression of IP_3_R1 is not altered between IRAG2-WT and IRAG2-KO. Our data show a significantly decreased basal Ca^2+^ release in pancreatic acinar cells from IRAG2-KO mice. Despite the lower basal Ca^2+^ levels in IRAG2-KO pancreatic acinar cells, upon stimulation with 1 µM of the muscarinic M3 receptor agonist carbachol, a higher amount of Ca^2+^ normalized to basal Ca^2+^ levels is released in IRAG2-KO pancreatic acinar cells compared to IRAG2-WT. However, maximum Ca^2+^ levels in these cells in response to stimulation with 1 µM carbachol are slightly lower in IRAG2-KO than in IRAG2-WT, though this effect is not significant. These effects are not observed for higher concentrations of carbachol. Therefore, our data suggest that IRAG2 leads to an activation of IP_3_ receptors under unstimulated, basal conditions. Loss of IRAG2 reduces the activity of the IP_3_ receptors and consequently diminishes basal intracellular Ca^2+^ levels and might therefore lead to a downregulation of IP_3_R2. This leads to the assumption that IP_3_R2 could be involved in basal Ca^2+^ release in pancreatic acinar cells. A reason for the enhanced Ca^2+^ release upon stimulation with 1 µM carbachol could be the upregulation of IP_3_R3 in the pancreas as a compensatory mechanism. It is shown that cytosolic Ca^2+^ concentration affects the activity of IP_3_ receptors in many cell types in a biphasic manner [26,27,28]. Therefore, another explanation could be that the lower basal Ca^2+^ levels in IRAG2-KO cells lead to enhanced activation of IP_3_ receptors upon stimulation and, therefore, to an increased amount of released Ca^2+^ normalized to basal Ca^2+^ levels. In IRAG2-KO pancreatic acinar cells, the frequency of Ca^2+^ oscillations is higher compared to IRAG2-WT cells. This may also be a mechanism to compensate for the lower intracellular Ca^2+^ levels in IRAG2-KO due to decreased activity of the IP_3_R to ensure an appropriate Ca^2+^ release after muscarinic stimulation. The effect of the different IP_3_ receptor subtypes and the extent of how they contribute to Ca^2+^ signaling and Ca^2+^ oscillations are still widely unknown [2]. In Hela cells and COS7 cells, it is reported that knockdown of IP_3_R1 leads to decreased total Ca^2+^ levels and termination of Ca^2+^ oscillations. In contrast, the knockdown of IP_3_R3 leads to more long-lasting Ca^2+^ oscillations, and it is shown that IP_3_R3 functions as an anti-oscillatory unit [2]. In B cells, which are genetically engineered to express only IP_3_R2, Ca^2+^ oscillations are more regular and long-lasting, whereas B cells that either express IP_3_R1 or IP_3_R3 showed rapidly damped Ca^2+^ oscillations [29]. These results are in contrast to the oscillatory signals we observe in pancreatic acinar cells of IRAG2-KO, where a knockdown of IRAG2 leads to an upregulation of IP_3_R3 and results in a higher frequency of oscillations. An explanation could be that IRAG2 modulates the IP_3_ receptor subtypes and, therefore, Ca^2+^ oscillations in pancreatic acinar cells, independent of the amount of expressed IP_3_R3. Hence, it is possible that IRAG2 leads to a stabilization of IP_3_R, leading to a decreased frequency of oscillations. In pancreatic acinar cells, it is suggested that IP_3_R3 may play a major role in the generation of agonist-induced Ca^2+^ oscillations [30,31,32]. This raises the possibility that modulation of IP_3_R3 by IRAG2 leads to a lower frequency of Ca^2+^ oscillations in WT cells. The fact that cytosolic Ca^2+^ concentration affects the activity of IP_3_ receptors in a biphasic manner [26,27,28] could be another reason for the increased frequency of Ca^2+^ oscillations in IRAG2-KO. It is possible that the lower basal Ca^2+^ levels of IRAG2-KO leads to an activation of IP_3_ receptors and followed by a higher frequency of Ca^2+^ oscillations in IRAG2-KO cells, maybe through IP_3_R3 but maybe also through other IP_3_ receptor subtypes with a higher affinity to cytosolic Ca^2+^, like IP_3_R1 or IP_3_R2 [29]. However, the fact that the expression of IP_3_R1 is rather low in the pancreas makes it unlikely that modulation of IP_3_R1 through IRAG2 contributes to Ca^2+^ oscillations in pancreatic acinar cells [25,32]. The downregulation of IP_3_R2 in the pancreas of IRAG2-KO may also contribute to the higher frequency of oscillations in IRAG2-KO acinar cells, as IP_3_R2 leads to more long-lasting and regular oscillations in B cells [29].

Release of Ca^2+^ and secretion of digestive enzymes like amylase are strongly linked, as the release of Ca^2+^ triggers secretion of amylase [1,18,19]. In our study, we also observe that reduced basal Ca^2+^ release leads to lower basal amylase secretion. Secretion of amylase upon stimulation with carbachol shows a tendency for a higher amount of secreted amylase normalized to basal secretion. This effect is not significant, but the tendency of increased amylase secretion is in accordance with the enhanced Ca^2+^ release upon stimulation when normalized to basal Ca^2+^ release. In our study, immunohistochemical investigation of amylase in the pancreas reveals a higher amount of amylase in pancreatic acinar cells of IRAG2-KO compared to IRAG2-WT, suggesting that more amylase remains in the cells, due to lower basal Ca^2+^ release, and therefore lower basal amylase secretion in IRAG2-KO acinar cells. Even if the amount of amylase is higher in IRAG2-KO cells compared to IRAG2-WT, we detect no accumulation of zymogen granules in IRAG2-KO cells compared to IRAG2-WT cells. It is reported that the double knockout of IP_3_R2 and IP_3_R3 causes exocrine dysfunction, leading to difficulties in nutrient digestion and decreased body weights of the double knockout mice [3]. IRAG2-KO mice show no differences in body weights until weaning; after weaning, IRAG2-KO mice reveal a tendency for decreased body weights. However, this effect is only temporary as body weight normalizes until day 45 after birth. This leads to the assumption that IRAG2-KO animals show almost normal nutrient digestion despite their lower basal amylase secretion. However, the accumulation of amylase in pancreata from IRAG2-KO mice could contribute to pancreatic diseases like pancreatitis. Therefore, IRAG2 could have a protective effect against pancreatic diseases. To determine the effect of IRAG2 on pancreatic diseases, further investigations using specific animal disease models would be needed.

In summary, with this work, we present new results about expression, molecular and physiological function of IRAG2:

1. IRAG2 is expressed in acinar cells of the murine exocrine pancreas and interacts with IP_3_ receptor types 1, 2, and 3 in the murine pancreas. Loss of IRAG2 leads to an upregulation of IP_3_R3 and to downregulation of IP_3_R2 expression in the pancreas.
2. Deletion of IRAG2 leads to lower basal Ca^2+^ release in murine pancreatic acinar cells, suggesting that IRAG2 leads to activation of IP_3_R under unstimulated basal conditions. However, IRAG2-KO reveals a higher amount of released Ca^2+^ normalized to basal release upon stimulation with 1 µM carbachol compared to IRAG2-WT. The frequency of Ca^2+^ oscillations is higher in IRAG2-KO pancreatic acinar cells compared to IRAG2-WT, maybe due to modulation of IP_3_ receptors through IRAG2.
3. Decreased basal Ca^2+^ release in IRAG2-KO acinar cells leads to lower basal amylase secretion. Lower release of amylase may cause a higher amount of amylase that remains in the zymogen granules of IRAG2-KO.

## 4. Materials and Methods

### 4.1. Animals

Global *Irag2* deficient mice were generated as described before [13]. The targeting vector contained the exon 8 of murine *Irag2* gene, which was flanked by a loxP-Neo cassette and loxP sites, sequences of the Flipase/FRT system (FRT) and the *lacZ* gene and was constructed by EUCOMM (Helmholtz Zentrum Munich, Neuherberg, Germany). “Floxed” mice containing this targeting vector were either mated with CMV Cre-mice (B6.C-Tg(CMV-cre)1Cgn/J, Jackson Laboratories), resulting in *lacZ* × *Irag2*^−/−^ mice expressing β-galactosidase, or mated with FLP-mice (TGM/hACTB-FLPe, kindly provided by Prof. Ralph Witzgall, University of Regensburg), followed by mating with CMV Cre mice, leading to *Irag2*^−/−^ mice not expressing β-galactosidase (Figure S1). Animals were bred and maintained in the animal facilities of the University of Regensburg according to the Guidelines of the Federation of European Laboratory Animal Science Associations (Bavaria, Germany; Regierung von Unterfranken: DMS 2532-2-326) with free access to food and water ad libitum, following to all guidelines according to the German animal protection law.

For all experiments, male and female IRAG2-KO (genotype: *Irag2*^−/−^), lacZ × IRAG2-KO (genotype: *lacZ* × *Irag2*^−/−^) and their WT littermates (IRAG2-WT; genotype: *Irag2*^+/+^, *Irag2*^fl/fl^) older than 8 weeks were used. For localization experiments, lacZ × IRAG2-KO mice were used, all other experiments were conducted with IRAG2-KO.

### 4.2. Tissue Preparation for Western Blot Analysis

Mice were euthanized by cervical dislocation, and pancreata were immediately removed, snap-frozen in liquid nitrogen, and then stored at −80 °C. Whole pancreas tissue or isolated pancreatic acinar cells were homogenized with 2% Lubrol-buffer (2% nonaethylene glycol mondodecyl ether, 150 mM NaCl, 20 mM Tris in ddH_2_O, pH 8.0), containing protease inhibitors (1 mM benzamidine, 0.5 µg × mL^−1^ leupeptin, 300 µM PMSF). The homogenate was centrifuged, and the supernatant was collected. Protein concentration was determined by a Lowry kit (Bio-Rad Laboratories, Inc., Munich, Germany), and samples were then stored at −80 °C.

### 4.3. Coimmunoprecipitation

Lysates (1000 µg) were incubated in coimmunoprecipitation-buffer (50 mM Tris HCl, 15 mM EGTA, 100 mM NaCl, 0.1% Triton X-100 in ddH_2_O, pH 7.5), containing protease inhibitors, 1× PhosSTOP (Roche, Mannheim, Germany), 1 mM DTT and 1 µg of anti-mouse LRMP (C-terminal) antibody = anti-IRAG2 antibody (Sigma-Aldrich^®^, Taufkirchen, Germany) for 90 min (min) on ice. After incubation, the mixture was centrifuged, and the supernatant was collected. Meanwhile, 15 µL of Protein-A-Sepharose-Beads (Sigma-Aldrich^®^, Taufkirchen, Germany) was washed with coimmunoprecipitation buffer three times, blocked with 3% BSA in coimmunoprecipitation-buffer for 30 min, following three washing steps with coimmunoprecipitation buffer. The supernatant was added to the washed and blocked beads and incubated overnight at 4 °C under rotation. After a three-time washing step, the beads were eluted with 2× Laemmli buffer. Proteins were analyzed by SDS-Page and Western Blot (4.4).

### 4.4. Western Blot Analysis

The expression of several proteins was analyzed by Western blotting. From 50 µg to 70 µg of protein was used for SDS-Page. After gel-run, proteins were blotted on a PVDF membrane (Immobilon^−^P, Merck KGaA, Darmstadt, Germany), followed by blocking the membrane in 5% non-fatty milk, containing 0.05% Tween^®^ 20 for 2 h (h) at room temperature (RT). Membranes were then incubated in primary antibodies (anti-mouse LRMP (C-terminal) = anti-IRAG2 (Sigma-Aldrich^®^, Taufkirchen, Germany): 1:1000; anti IP_3_R1 (Novusbio, Abingdon, UK): 1:1000; anti IP_3_R2 (Santa Cruz, Heidelberg, Germany): 1:200; anti-IP_3_R3 (BD Transduction Laboratories^TM^, BD Biosciences, San Jose, USA): 1:100; anti-β-Galactosidase (Abcam plc, Cambridge, UK): 1:1000; anti IRAG1 (in house production): 1:200) at 4 °C overnight. After incubation in HRP-conjugated secondary antibodies (anti-mouse (Sigma-Aldrich^®^, Taufkirchen, Germany): 1:10,000; anti-rabbit (Dianova GmbH, Hamburg, Germany): 1:10,000; anti-goat (Dianova GmbH, Hamburg, Germany): 1:10,000) for 2 h at RT, the detection was carried out by Clarity^TM^ Western ECL Substrate (Bio-Rad Laboratories, Inc., Munich, Germany) and ChemiDoc^TM^ MP Imaging System (Bio-Rad Laboratories, Inc., Munich, Germany). The intensity of protein bands was normalized to the total protein signal of each lane using stain-free technology. Therefore, 1.5% trichloroethanol-containing handcasting SDS-gels were used. Quantification was carried out using the Image Lab^TM^ Software (Bio-Rad Laboratories, Inc., Munich, Germany).

### 4.5. Preparation of Dispersed Mouse Pancreatic Acinar Cells

Freshly dispersed acini were prepared by methods described before [33]. Briefly, pancreata were excised and acini isolated by enzymatic digestion with 50 U/mL units Collagenase 2A (Worthington Biochemical Corporation, Lakewood, NY, USA). Acini were then filtered through a 100 µm Nylon-mesh, purified by sedimentation through 4% BSA in buffer 2 times and resuspended in a HEPES-Ringer-buffer (20 mM Hepes, 95 mM NaCl, 4.7 mM KCl, 0.6 mM MgCl_2_, 1.3 mM CaCl_2_, 10 mM Glucose, 1× non-essential amino-acids-solution (Sigma-Aldrich^®^, Taufkirchen, Germany), 2 mM L-glutamine, pH 7.4), containing 1 mg × mL^−1^ BSA and 0.1 mg × mL^−1^ soybean trypsin inhibitor (Santa Cruz, Heidelberg, Germany). Before and between all steps, the medium was gassed with 5% CO_2_/ 95% O_2_.

### 4.6. Measurement of Intracellular Calcium

Freshly isolated acini were embedded in Matrigel (Corning, Amsterdam, The Netherlands); 10 µL of the cells-suspension in Matrigel were transferred on a coverslip, placed in the cave of a 12-well plate, and covered with 1 mL Ringer´s solution (139 mM NaCl, 0.4 mM KH_2_PO_4_, 1.6 mM K_2_HPO_4_⋅3 H_2_O, 5 mM glucose, 1 mM MgCl_2_⋅6 H_2_O, 1.3 mM Ca-gluconate⋅1 H_2_O, pH 7.4). After embedding, cells were allowed to rest for 30 min and then loaded with 2 µM Fura-2-AM (Biomol, Hamburg, Germany) and 0.02% Pluronic F-127 (Invitrogen, Darmstadt, Germany) for 90 min at RT. Cells were perfused with Ringer’s solution at 37 °C in an imaging chamber, and fluorescence was detected using an Axiovert S100 inverted microscope (Zeiss, Oberkochen, Germany) and a high-speed polychromator system (VisiChrome, Puchheim, Germany). Fura-2 was excited at 340/380 nm, and emission was recorded between 470 nm and 550 nm using a CoolSnap HQ CCD camera (Roper Scientific, Planegg, Germany/Visitron Systems, Puchheim, Germany). Data were evaluated using the MetaFluor software (Universal Imaging, New York, NY, USA).

### 4.7. Amylase Secretion Assay

Amylase secretion assay was performed as described before [34]. Freshly isolated pancreatic acinar cells were preincubated for 30 min at 37 °C. 1 mL aliquots of cell suspension were stimulated with 1 µM or 10 µM carbachol (Sigma-Aldrich^®^, Taufkirchen, Germany) for 30 min in a shaking water bath at 50 rpm. After incubation, the acinar cell suspension was centrifuged for 30 s (sec) at 5000× *g*, and the supernatant was assayed for Amylase using the Phadebas^®^ Amylase test (Phadebas AB, Kristianstad, Sweden). Results were expressed as a percentage of initial acinar amylase content.

### 4.8. X-Gal-Staining

For localization of IRAG2, an X-Gal-staining for β-galactosidase activity was performed on cryosections. Cryoprotection of excised pancreata from IRAG2-WT and lacZ×IRAG2-KO was performed according to previous protocols, with some modifications [35]. Pancreata were fixed in 2% PFA solution overnight at 4 °C, following cryoprotection by a series of Sucrose-solutions in PBS with increasing concentrations (5%, 10%, 20%). Finally, pancreata were embedded in Tissue-Tek^®^ Mounting Medium (A. Hartenstein GmbH, Würzburg, Germany) and frozen at −80 °C for at least 24 h. Then, 8 µm cryosections were performed using Superfrost Plus^®^ slides (Thermo Scientific, Braunschweig, Germany) and a Cryotome (Leica Biosystems, Wetzlar, Germany). X-Gal staining was carried out according to previous protocols, with some modifications [36,37,38]. Sections were thawed 30 min before staining, fixed with ice-cold acetone for 10 min, and permeabilized with 0.3% Triton-X 100 in PBS for 30 min. After incubation with X-Gal working solution (1 mg × mL^−1^ X-Gal (GERBU Biotechnik GmbH, Heidelberg, Germany), 5 mM K_4_[Fe(CN)_6_]∙3H_2_O, 5 mM K_3_[Fe(CN)_6_], 2 mM MgCl_2_ in PBS, pH 7.4) for 20 h at 37 °C, slides were counterstained with Nuclear Fast Red solution (Merck KGaA, Darmstadt, Germany) and embedded in Dako Glycergel Mounting Medium (Agilent, Waldbronn, Germany). Images were taken with an Axiovert 200 microscope (Carl Zeiss AG, Jena, Germany) using the AxioVision software (Carl Zeiss AG, Jena, Germany).

### 4.9. Immunohistochemistry

Pancreata were removed, incubated in a 3% PFA solution (3% PFA, 1 mM EGTA, 15 mM K_2_HPO_4_∙3H_2_O, 2 mM MgCl_2_∙6H_2_O, 90 mM NaCl, 100 mM sucrose in ddH_2_O, pH 7.4) at 4 °C overnight and embedded in paraffin wax according to previous protocols [39], and 2.5 µM sections were performed with a microtome (Thermo Scientific, Braunschweig, Germany) by using polylysine-slides (Thermo Scientific, Braunschweig, Germany). For immunohistochemistry experiments, sections were dewaxed and rehydrated (2 × 10 min xylene, 3 × 5 min isopropanol, 2 × 5 min 100% methanol, 2 min ddH_2_O), antigen retrieval was performed by boiling the slides in a Tris/EDTA solution (1.2 g × L^−1^ Tris, 0.372 g × L^−1^ EDTA, pH 8.5) for 30 min. Sections were blocked with 10% Horse Serum (Sigma-Aldrich^®^, Taufkirchen, Germany) in 1% BSA PBS for 2 h at RT, incubated with primary-antibody (anti-Amylase (Santa Cruz, Heidelberg, Germany): 1:200) at 4 °C overnight, following incubation with the appropriate fluorophore-coupled secondary antibody (Alexa Fluor^®^ 647 anti-mouse (Invitrogen, Eugene, OR, USA): 1:200) for 2 h at RT in the dark. Slides were washed 3 × 5 min with PBS, mounted in Dako Glycergel Mounting Medium (Agilent, Waldbronn, Germany), and fluorescence was detected with an Axiovert 200 microscope (Carl Zeiss AG, Jena, Germany) using the AxioVision software (Carl Zeiss AG, Jena, Germany). Assessment of fluorescence was carried out with the MetaMorph^®^ Software 7.10.4 (Molecular Devices, San Jose, CA, USA), and fluorescence in IRAG2-KO was normalized to IRAG2-WT. Results were given as x-fold change of expression in IRAG2-KO normalized to IRAG2-WT.

### 4.10. Statistical Analysis

All data are shown with mean ± SEM. The Shapiro–Wilk test was used to test the samples for normality. For normally distributed parameters, an unpaired Student´s t-test was used to calculate significant differences between two means when variances between the groups were not different. Non-parametric data were analyzed using the Wilcoxon–Mann–Whitney test. Statistical analysis was performed using “GraphPad Prism version 5.01”. Significant differences in the graphs are shown by asterisks (*) (*p* < 0.05), (**) (*p* < 0.01), and (***) (*p* < 0.001).

## Data Availability

The datasets for this manuscript are not publicly available because the raw data supporting the conclusions of this manuscript will be made available by the authors, without undue reservation, to any qualified researcher. Requests to access the datasets should be directed to the corresponding author jens.schlossmann@chemie.uni-regensburg.de.

## Supplementary Materials

The following are available online at https://www.mdpi.com/article/10.3390/ijms222413409/s1.
