# Supplementary material for: IRAG2 Interacts with IP3-Receptor Types 1, 2, and 3 and Regulates Intracellular Ca2+ in Murine Pancreatic Acinar Cells
Source: Int J Mol Sci. 2021 Dec 14;22(24):13409. doi: 10.3390/ijms222413409 (PMC8707672; doi:10.3390/ijms222413409)
Supplement: Supplementary file 1 [file ijms-22-13409-s001.zip › ijms-1501892-supplementary.pdf]

Supplementary Material

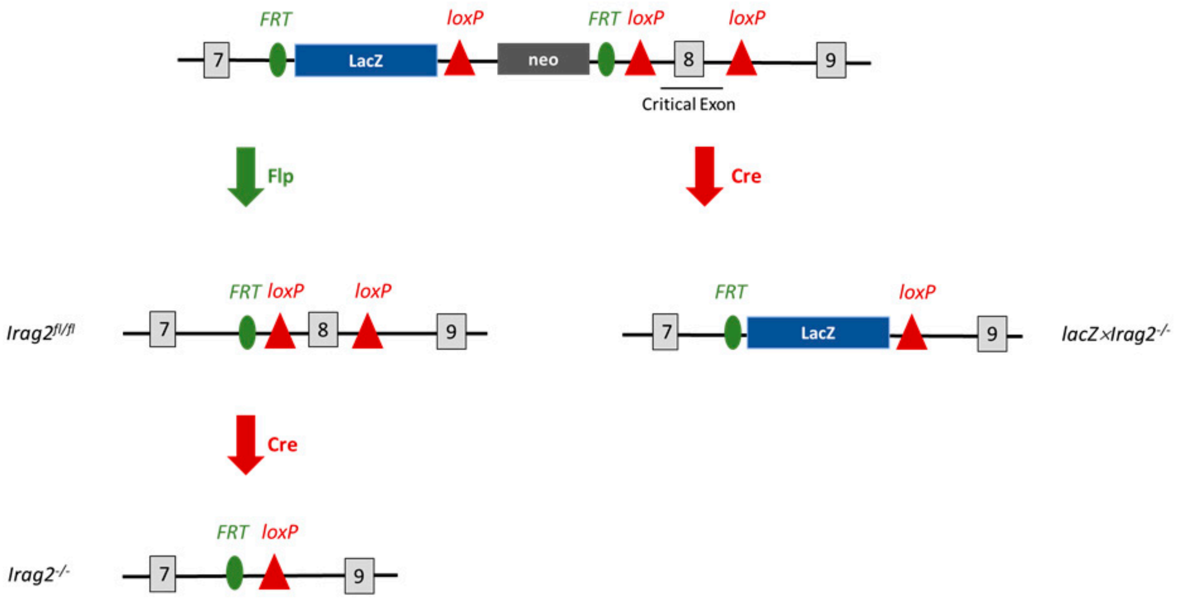

Figure S1. Schematic illustration of generation of IRAG2-KO and lacZ×IRAG2-KO mice. Vector contains exon 8 of murine *Irag2* gene, that is flanked by a loxP-Neo cassette and loxP sites, sequences of the Flipase/FRT system (FRT) and the *lacZ* gene. Mice carrying the vector were merged with Flp mice resulting in *Irag2*$^{fl/fl}$ mice followed by breeding with CMV Cre-mice which yielded *Irag2*$^{-/-}$ mice (left panel). When mice with the vector were bred at first with CMV Cre-mice the *lacZ×Irag2*$^{-/-}$ emerged (right panel).

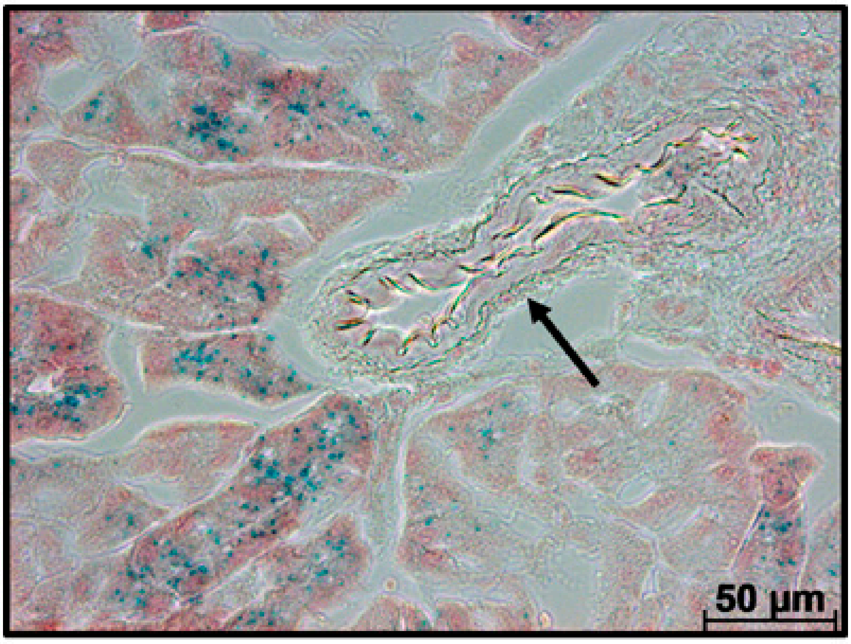

lacZ×IRAG2-KO

Figure S2. X-Gal staining of pancreatic ducts. X-Gal staining showed expression of β-galactosidase as a reporter for IRAG2 in pancreatic acinar cells of lacZ×IRAG2-KO mice, no expression was detectable in ducts (indicated by arrow).

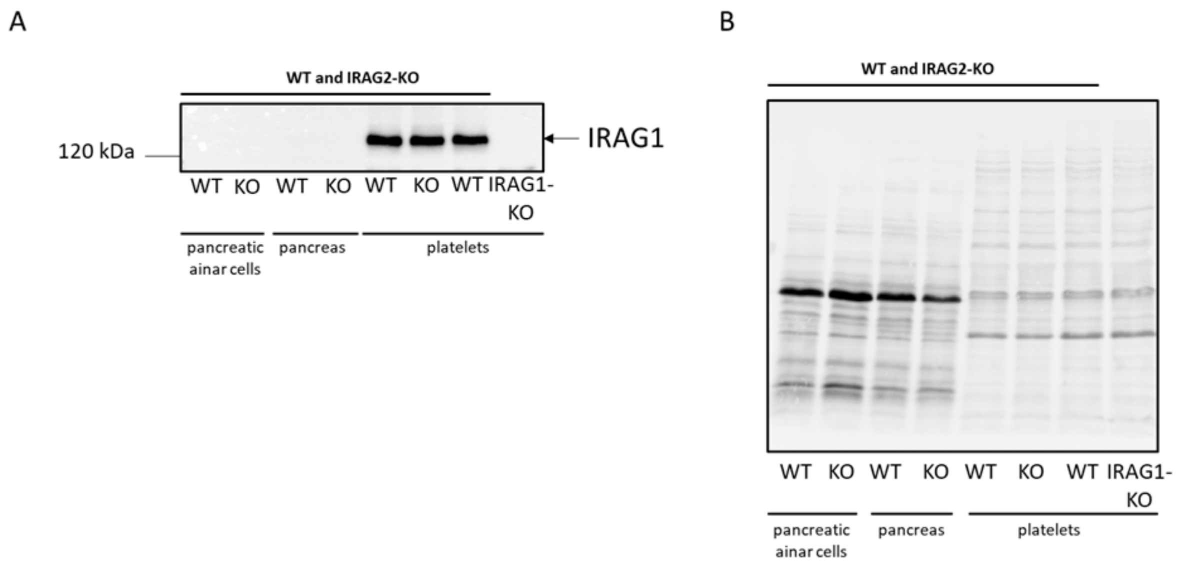

Figure S3. Expression of IRAG1 in murine pancreas. (**A)** Western blot analysis of IRAG1 expression reveals no signal for o IRAG1 in pancreatic acinar cells or whole pancreas tissue of IRAG2-KO (KO) and WT (WT) mice. As positive control for IRAG1 expression platelets from IRAG2-WT and IRAG2-KO mice, and as IRAG1 negative control platelets of IRAG1-KO mice were used. (**B**): Total protein for loading control. 70 µg protein was applied per lane.

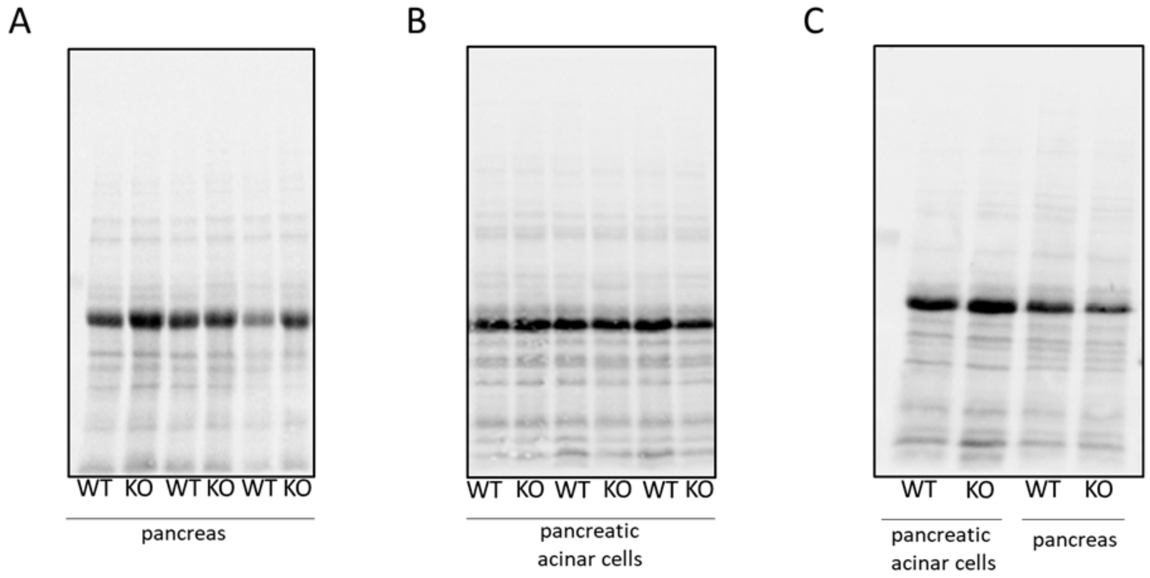

Figure S4: Total protein images of whole pancreata **(A, C)** and pancreatic acinar cells **(B, C).**
Total protein images from western blots for the analysis of IRAG2 expression in murine pancreas or pancreatic acinar cells IRAG2-WT (WT) and IRAG2-KO (KO) mice (Figure 1A,C) and β-galactosidase expression in whole murine pancreas or pancreatic acinar cells from IRAG2-WT (WT) and lacZ×IRAG2-KO (KO) mice (Figure 1E). Input per lane: 70 µg protein from whole pancreas lysates and 50 µg protein from pancreatic acinar cells.

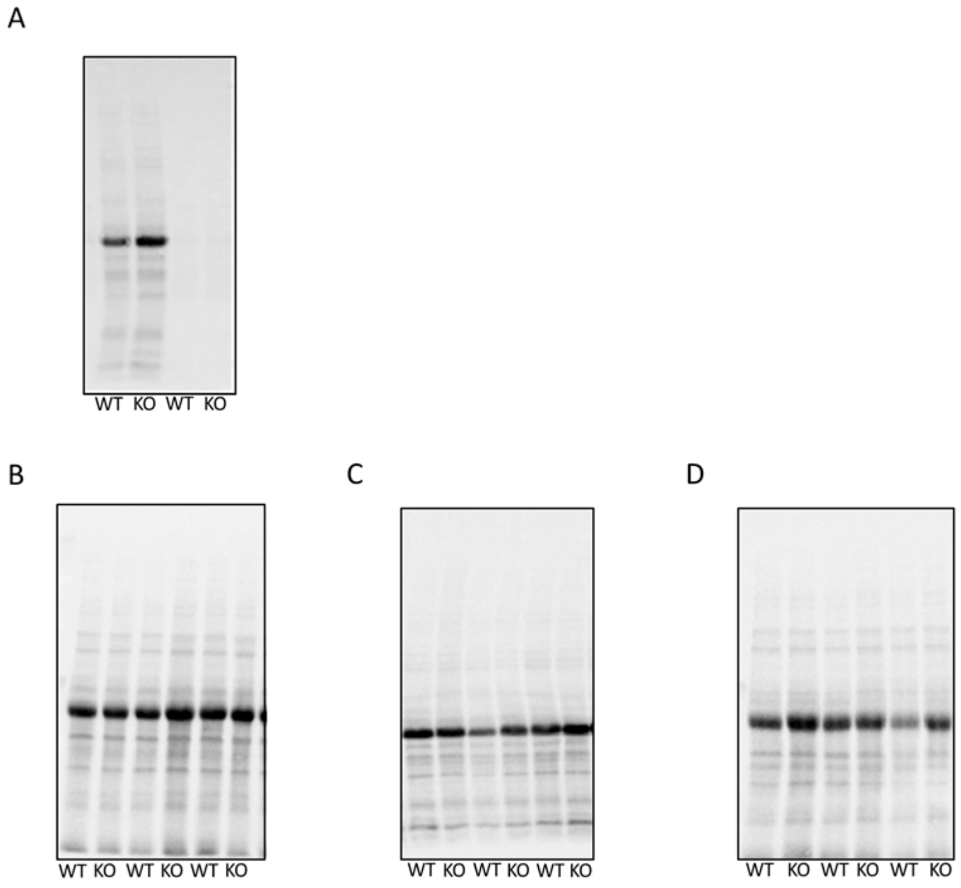

Figure S5: Total protein images of pancreata from coimmunoprecipitation **(A)** and analysis of IP$_3$R3 **(B)**, IP$_3$R2 **(C)** and IP$_3$R1 **(D)** expression.
Total protein images from western blots for coimmunoprecipitation (Figure 2A) and analysis of IP$_3$R3 expression (Figure 2B), IP$_3$R2 expression (Figure 2D) and IP$_3$R1 expression (Figure2F) in IRAG2-WT (WT) and IRAG2-KO (KO) mice. Input per lane: 70 µg protein. For coimmunoprecipitation 1500 µg murine pancreas lysate was used.

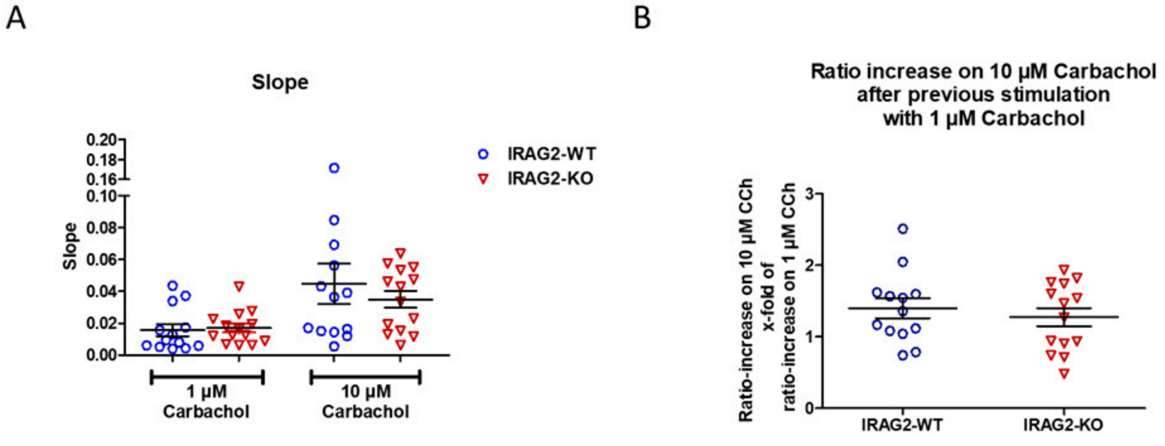

Figure S6. (**A**) Slopes of Ca^{2+} release curves upon stimulation with 1 µM carbachol and 10 µM were calculated and revealed no differences between IRAG2-WT and IRAG2-KO (WT: n=13 experiments from 5 animals, KO: n=14 experiments from 5 animals). (**B**) After stimulation with 1 µM carbachol, cells were stimulated with 10 µM carbachol. Neither IRAG2-WT nor IRAG2-KO showed reduced stimulatability with 10 µM carbachol after previous stimulation with lower carbachol concentration (WT: n=13 experiments from 5 animals, KO: n=14 experiments from 5 animals). Circles indicate the individual value of each experiment in IRAG2-WT and triangles show individual values for each experiment from IRAG2-KO. The numbers in the graphs indicate the mice that were investigated and mean ± SEM are shown.

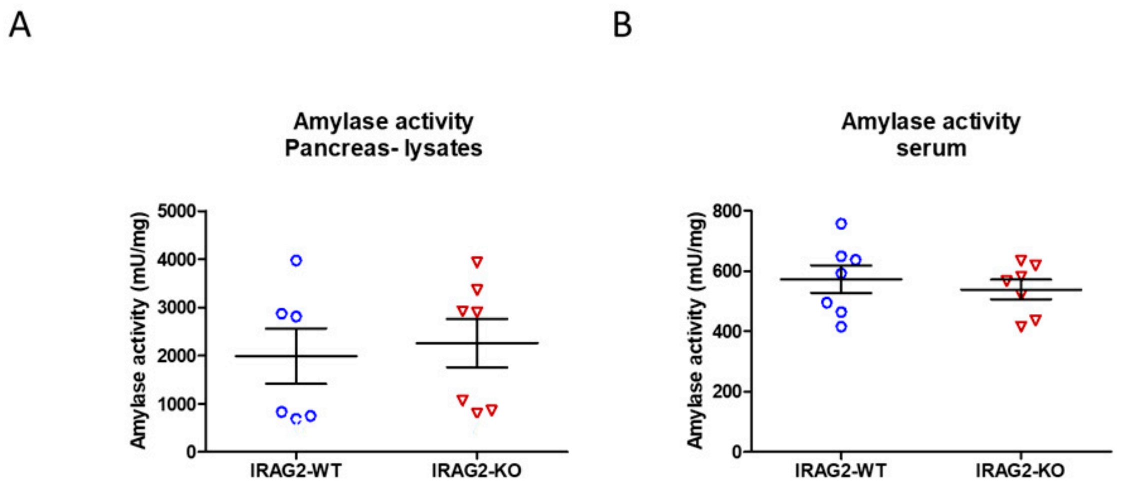

Figure S7. Amylase activity in IRAG2-WT and IRAG2-KO pancreas lysates and serum probes. (**A-B**) Amylase activity assay was performed on pancreas lysates (WT: n=6; KO: n=7) (**A**) and serum probes (WT: n=7, KO: n=7) (**B**) from IRAG2-WT and IRAG2-KO mice, with no difference in amylase activity. For determination of amylase activity an Amylase Assay Kit (Colorimetric) (ab102523, Abcam plc, Cambridge, UK) was used. Circles indicate the individual value of each experiment in IRAG2-WT and triangles show individual values for each experiment from IRAG2-KO. The numbers in the graphs indicate the mice that were investigated and mean ± SEM are shown.

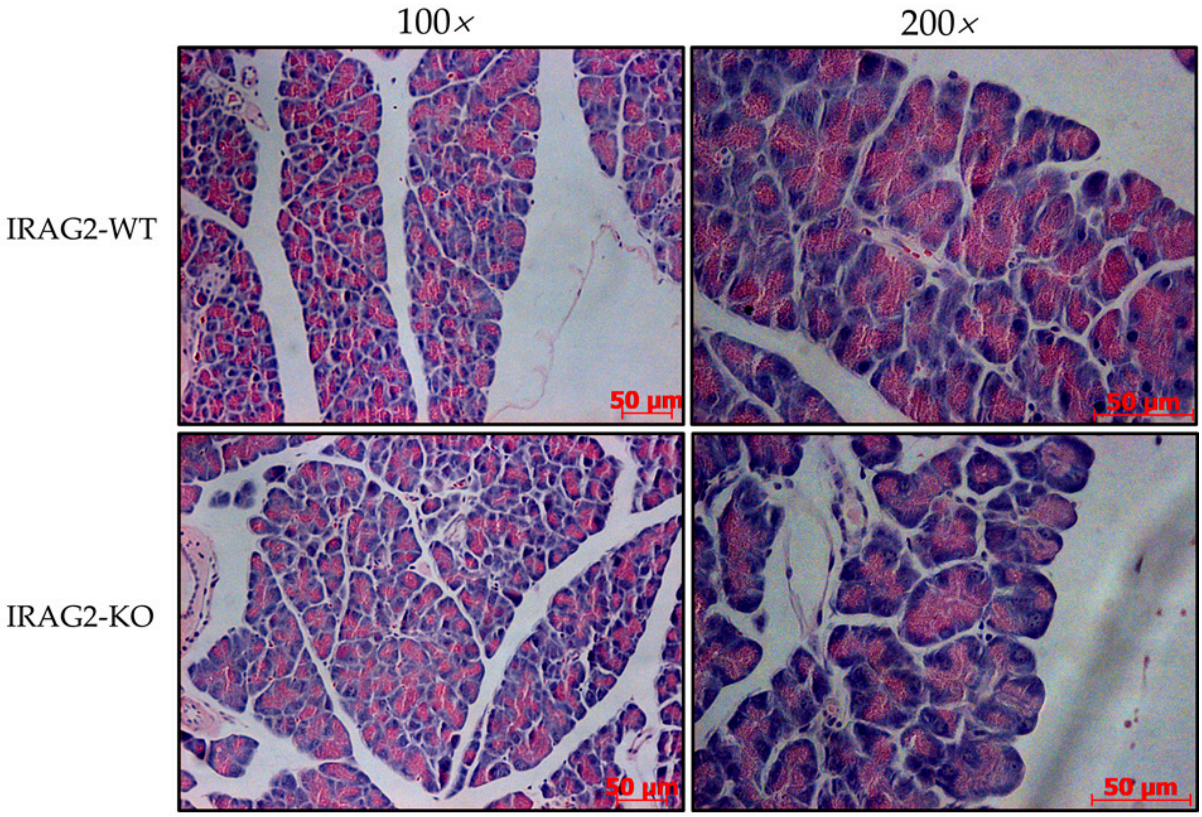

Figure S8. Hematoxylin-Eosin-staining (HE-staining) of WT and IRAG2-KO pancreata showed no difference between IRAG2-WT and IRAG2-KO in morphology or in amount of zymogen-granules. Images left: magnification 100×; images right: magnification 200×.

For HE-staining pancreata were embedded as described in 4.9. 2.5 µm sections were performed, dewaxed and rehydrated (2 × 10 min xylene, 3 × 5 min isopropanol, 2 × 5 min 100% methanol, 2 min ddH_2O). Slides were incubated in hematoxylin solution (Carl Roth® GmbH, Karlsruhe, Germany) for 20 sec, washed in ddH_2O for 10 sec and dipped in 0.1% ammonium-hydroxide solution for 2 times. After washing (2 × 2 min ddH_2O), slides were stained in eosin solution (Sigma-Aldrich®,Taufkirchen, Germany) for 1.5 min, washed (5 × 2 min ddH_2O) and treated with 80% ethanol for 3 min. Slides were dehydrated again (3 min 100% ethanol, 5 min xylene) and mounted in DePeX® (Serva GmbH, Heidelberg, Germany).

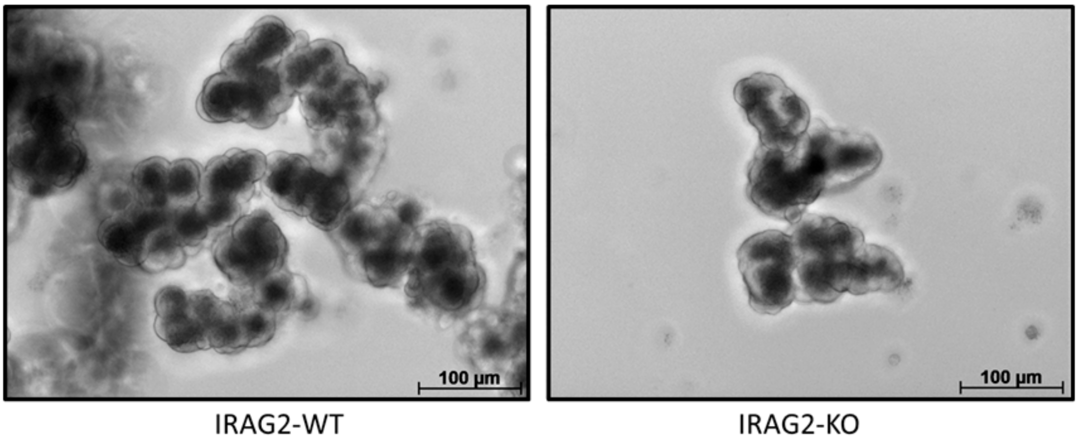

Figure S9. Image of pancreatic acinar cells after isolation, with no morphological difference between IRAG2-WT and IRAG2-KO acinar cells (magnification 200×).

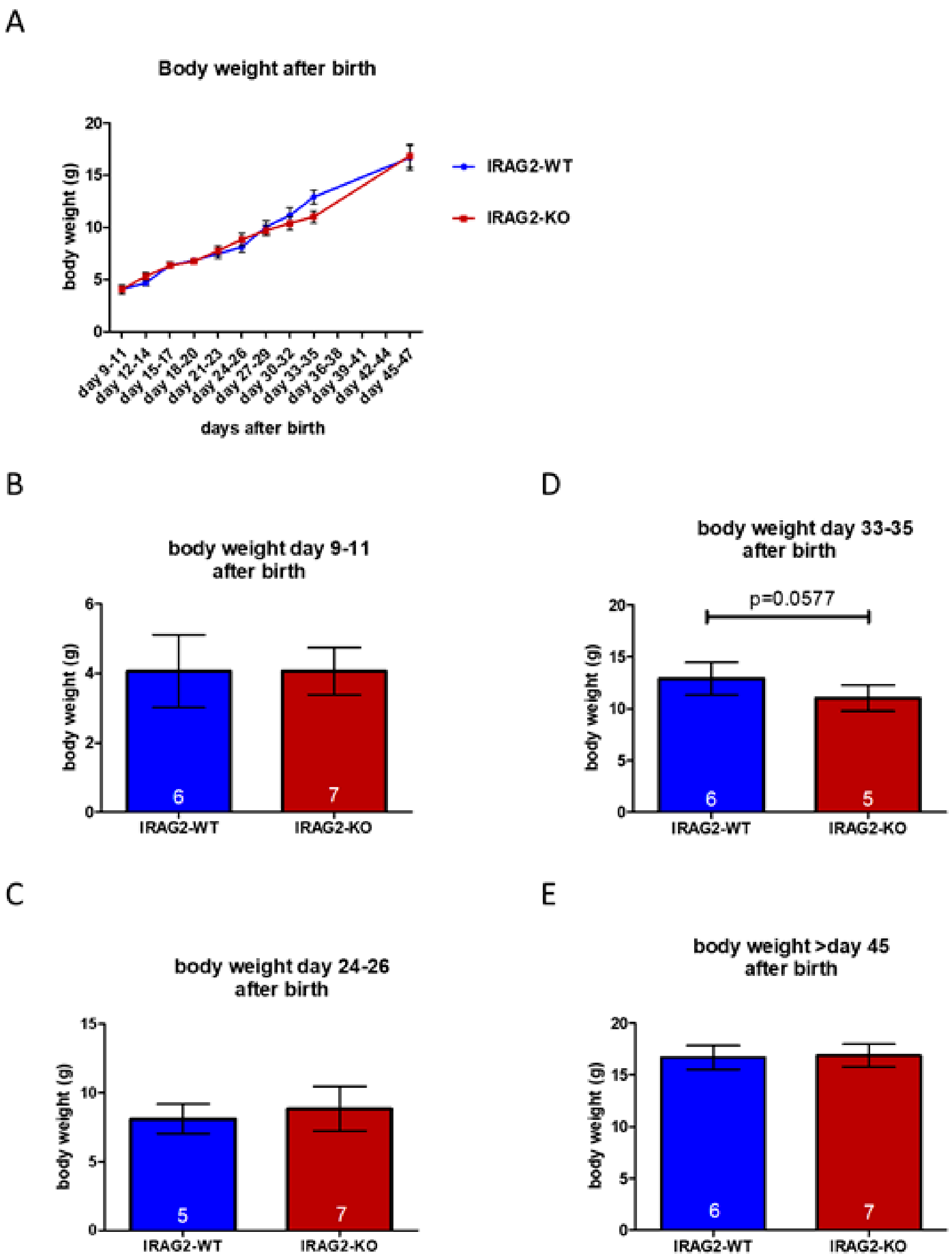

Figure S10. (**A**) body weights of WT and IRAG2-KO animals starting on day 9 after birth until day 45-47 after birth. (**B-C**) Body weights 9-11 days (**B**) and 24-26 days after birth (**C**) revealed no differences between WT and IRAG2-KO. (**D**) On days 33-35 IRAG2-KO showed a tendency for a lower body weight. (**E**) After day 45 no difference between IRAG2-WT and IRAG2-KO was apparent. The numbers in the graphs indicate the mice that were investigated and mean ± SEM are shown.

For body weight data, IRAG2-WT and IRAG2-KO mice were marked with an earclip at day 10 after birth and weighed every second day until day 45-47.
